# Involvement of heat shock proteins on Mn-induced toxicity in *Caenorhabditis elegans*

**DOI:** 10.1186/s40360-016-0097-2

**Published:** 2016-11-02

**Authors:** Daiana Silva Avila, Alexandre Benedetto, Catherine Au, Julia Bornhorst, Michael Aschner

**Affiliations:** 1 Federal Pampa University, Uruguaiana, RS Brazil; 2 Faculty of Health and Medicine, Division of Biomedical and Life Sciences, Lancaster University, Lancaster, UK; 3 Institute of Healthy Ageing, and Department of Genetics, Evolution and Environment, University College London, London, UK; 4 Department of Food Chemistry, Institute of Nutritional Science, University of Potsdam, Potsdam, Germany; 5 Department of Molecular Pharmacology, Albert Einstein College of Medicine, Forchheimer 209, 1300 Morris Park Avenue, Bronx, NY 10461 USA; 6 Center for Molecular Toxicology, and Vanderbilt Kennedy Center for Research on Human Development, Vanderbilt University School of Medicine, Nashville, TN USA

**Keywords:** *Caenorhabitis elegans*, Manganese, Heat shock proteins, *hsp-70*, *pink1*

## Abstract

**Background:**

All living cells display a rapid molecular response to adverse environmental conditions, and the heat shock protein family reflects one such example. Hence, failing to activate heat shock proteins can impair the cellular response. In the present study, we evaluated whether the loss of different isoforms of heat shock protein (*hsp*) genes in *Caenorhabditis elegans* would affect their vulnerability to Manganese (Mn) toxicity.

**Methods:**

We exposed wild type and selected *hsp *mutant worms to Mn (30 min) and next evaluated further the most susceptible strains. We analyzed survival, protein carbonylation (as a marker of oxidative stress) and Parkinson’s disease related gene expression immediately after Mn exposure. Lastly, we observed dopaminergic neurons in wild type worms and in *hsp-70* mutants following Mn treatment. Analysis of the data was performed by one-way or two way ANOVA, depending on the case, followed by post-hoc Bonferroni test if the overall *p* value was less than 0.05.

**Results:**

We verified that the loss of *hsp-70, hsp-3 and chn-1* increased the vulnerability to Mn, as exposed mutant worms showed lower survival rate and increased protein oxidation. The importance of *hsp-70* against Mn toxicity was then corroborated in dopaminergic neurons, where Mn neurotoxicity was aggravated. The lack of *hsp-70* also blocked the transcriptional upregulation of *pink1*, a gene that has been linked to Parkinson’s disease.

**Conclusions:**

Taken together, our data suggest that Mn exposure modulates heat shock protein expression, particularly HSP-70, in *C. elegans*. Furthermore, loss of *hsp-70* increases protein oxidation and dopaminergic neuronal degeneration following manganese exposure, which is associated with the inhibition of *pink1* increased expression, thus potentially exacerbating the vulnerability to this metal.

## Background

Molecular chaperones are highly evolutionarily conserved and ubiquitously found in subcellular compartments, cells, and tissues, being essential for the stability of the proteome under normal and stressful conditions [1]. The expression of many molecular chaperones is regulated by environmental and physiological stresses that can interfere with folding stability, leading to a flux of misfolded proteins [2]. Stress responsive molecular chaperones are referred to as heat shock proteins (HSPs) and classified by gene families according to their molecular mass as Hsp100, Hsp90, Hsp70, Hsp60, Hsp40 and small Hsps (sHsps). HSPs exert their physiological effect by assisting the formation of new proteins as well as by preserving existing structures. However, they also display major functions in pathological conditions, especially through structural rectification of denatured proteins and solubilization of protein aggregates carrying them on to the proteasome system [2, 3].

Metal exposure at different levels can cause oxidative stress, which can lead to protein aggregation [4, 5]. Thereby, metals themselves are able to generate aberrant interactions with proteins such as beta-amyloid, α synuclein and prion proteins [5, 6]. In this context, Manganese (Mn) poisoning has been associated with increased heat shock protein levels, especially HSP70 [7–9]. Mn is widely used in industry and in agriculture, being found in several products such as batteries, pesticides, gasoline, parenteral nutrition, water purification agents and drugs [10–14]. As a consequence, exposed subjects may develop a syndrome known as manganism, where alterations in movement, speech and face expression may appear [14–16].

Manganism and Parkinson´s disease (PD) share several symptoms and molecular mechanisms [17]. Several lines of evidence point out that the behavioral and cognitive impairments are due to the dopaminergic alterations in brain areas that are involved in the movement circuitry. Depletion of dopamine (DA) from the dopaminergic (DAergic) neurons, mitochondrial dysfunction, oxidative stress and neuronal death have been reported in both disorders. While PD is mostly idiopathic in its etiology, many genes have now been associated with the disease called as PD-related genes. The mutation of dj-1, pink-1, parkin, for example, have been strongly linked to the early-onset of PD neurodegenerative disease in humans. In addition, it has been demonstrated that mutation in these genes also relate to onset of manganism [18–20]. As a putative treatment, recently, studies provided evidence on the important role of HSP70 in recovering DAergic neurons or degrading misfolded proteins in PD models [21, 22].

In order to study the involvement of HSPs on Mn-induced neurotoxicity, we used the *Caenorbabditis elegans* model. This nematode incorporates 302 neurons, 8 of them being dopaminergic. Its transparent body and ease of genetic manipulability turns it into very interesting model to study neurodegeneration and to unravel molecular targets of toxicants. Our group has already demonstrated that Mn causes specific degeneration in the dopaminergic neurons through molecular mechanisms that replicate the effects observed in mammalian models. Furthermore, *C. elegans* possess 21 isoforms of HSP, which have been shown to function as chaperones and to have antioxidant role in worms as well as in mammals [23, 24].

Hence, we hypothesized that the deletion of hsp genes would increase Mn- induced oxidative stress and DAergic neurotoxicity. Furthermore, we investigated whether this deletion would affect the expression of some PD-related genes, based on the hypothesis that HSPs might be carriers of DJ1, PDR1 and PINK1 to the mitochondria following Mn stress.

## Methods

### Chemicals

Oxyblot protein oxidation analyses kits were purchased from Millipore (S7150- Billerica, CA). All the other reagents were obtained from Sigma (St Louis, MO).

### C. elegans *strains and handling of the worms*

*C. elegans* Bristol N2 (wild type) PS3551 (*hsf-1(sy441)I*), BR2823 (*chn-1(by155)I*), RB1104 (*hsp-3(ok1083)X*), LL 1009 (*daf-21*(*nr2081*)/nT1 [unc-?(*n754*) let-?] IV;V), RB825 (*hsp-43(ok647*)X), VC281 (*hsp-12.6(gk156) IV*), VC1099 (*hsp-4(gk514)II*), CNH-1 gf (gain-of-function) were handled and maintained at 20 °C on *E. coli* OP50/ NGM (nematode growth media) plates as previously described [25]. These strains were provided by the Caenorhabditis Genetics Center (CGC, Minnesota). *hsp-70 (tm2318) I*, was a gift from the Mitani lab. Synchronous L1 population were obtained by isolating embryos from gravid hermaphrodites using bleaching solution (1 % NaOCl; 0.25 M NaOH), followed by floatation on a sucrose gradient to segregate eggs from dissolved worms and bacterial debris, accordingly to standard procedures previously described [26].

### Dose–response curves after Mn exposure

Five thousand synchronized L1 stage worms per dose were treated for 30 min with each of the compounds, followed by three washes with 85 mM NaCl soultion. Worms were placed on OP50 seeded NGM plates and the dose–response curves were plotted from scoring the number of surviving worms on each dish at 24 h post-exposure. Dose response curves and LD_50_ values were obtained from those curves. Worms were then exposed for 30 min to 35 mM manganese chloride (MnCl_2_), which corresponds to the LD_25_ for MnCl_2_ as previously reported by Benedetto et al. [27]. For all dose–response curves, scores were normalized to percent control (0 mM MnCl_2_ exposure).

### Protein oxidation determination

Twenty thousand worms were exposed to MnCl_2_ (3, 10, 20, 35, 50 mM), as previously described. Next, worms were homogenized by sonication in a lysis buffer containing 85 mM sodium chloride, 1 % Triton X-100, 10 mM Tris Buffer (pH 6.8), 1× protease inhibitor and 50 mM dithiotreitol (DTT). After centrifugation (11,000xg for 1 min), the supernatant was isolated and protein concentration was determined with the Bradford method [28]. One hundred micrograms of proteins were derivatized with 2,4,dinitrophenylhydrazine (DNPH), which is converted to 2,4, dinitrophenylhydrazone (DNP) in the presence of carbonyls from oxidized proteins. The carbonyls were detected by western blotting with a commercial antibody directed against derivatized carbonyl groups (anti 2,4- DNP, rabbit IgG), and visualized by horseradish peroxidase conjugated secondary antibody according to the kit instructions (Oxyblot analysis kit, Millipore). Purified β-actin (A1978, Sigma, St. Louis, MO) was used as a control and the bands’ density was acquired with Image J (Rasband, W.S., ImageJ, U. S. National Institutes of Health, Bethesda, Maryland, USA, http://imagej.nih.gov/ij/, 1997–2011.).

### Confocal microscopy

For each slide, at least 20 worms were mounted on 4 % agarose pads in M9 and anaesthetized with 0.2 % tricaine/0.02 % tetramisole in M9. Fluorescence observations were performed with an epifluorescence microscope (Nikon Eclipse 80i, Nikon Corporation, Tokyo, Japan) equipped with a Lambda LS Xenon lamp (Sutter Instrument Company) and Nikon Plan Fluor 20× dry and Nikon Plan Apo 60 × 1.3 oil objectives. Microscopes were housed in air-conditioned rooms (20–22 °C). Worms were observed 2 h after Mn exposure.

### Real time PCR

Total RNA was isolated using the TRIzol reagent (Invitrogen) and the RNeasy mini kit (Qiagen). First-strand cDNA synthesis was performed with an equal amount of RNA using the Thermoscript real-time PCR kit (Invitrogen) as per the kit's instructions. The genes observed through real-time PCR were as follows: *hsp-70, pdr-1, dj-1, parkin* (Table 1)*.* The housekeeping gene *act-*1 was used as an internal control. Primer sequences are available on request. mRNA expression was quantified using the SYBR green detection method on an Bio-Rad real-time PCR system. Relative quantification for the expressed genes was done using the comparative *C*_T_ (ΔΔ*C*_T_) method.

**Table 1:** List of primers used in this study

Gene	Seq 5' to 3'- Forward	Seq 5' to 3' -Reverse
hsp-70	CCATGACTTAGTGGGACAAC	AAGACTACGCCTTCCTACGT
pink1	TCATGTCTCGCTGAGCAACT	GGCTCCATATCCGAATGCT
djr1.1	CTCGTGGTGAAATTCGTGTG	GCGGACAAGTAGGCTTTCAG
pdr1	CAAATGTCTAGCCTGCAACG	CGAACTATTGCACCCTGGAT
act1	ATCACCGCTCTTGCCCCATC	GGCCGGACTCGTCGAATTCTTG

### Statistics

Dose–response lethality curves, longevity curves and ROS content and oxyblot analysis were generated with GraphPad Prism (GraphPad Software Inc.). We used a sigmoidal dose–response model with a top constraint at 100 % to draw the curves and determine the LD_50_ or the average lifespan values reported in the graphs. Statistical analysis of significance was carried out by one-way or two way ANOVA, depending on the case, followed by post-hoc Bonferroni test if the overall p value was less than 0.05. In all figures, error bars represent the standard errors of the mean (SEM).

## Results

**Fig. 1:**
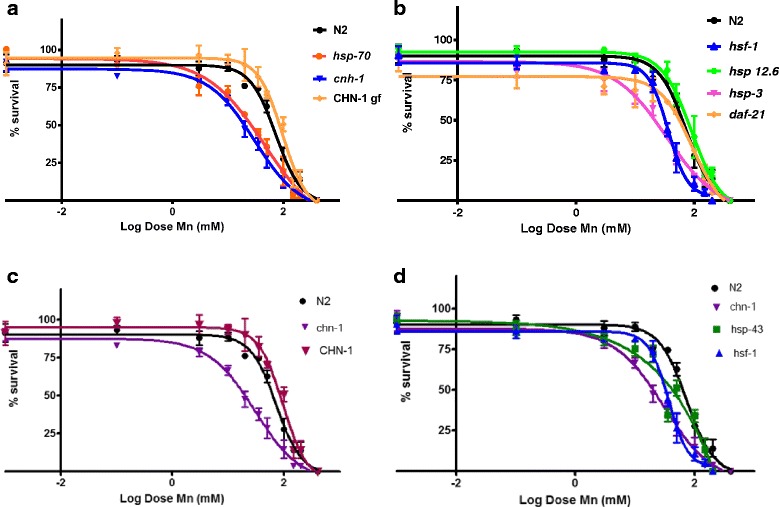
Dose–response curves for acute treatment with Mn (30 min) in different *hsp* mutants, all compared to N2 (wildtype). **a** N2, *hsp-70, hsp-4, hsp-12.6*; (**b**) N2, *hsp-3, daf-21*; (**c**) N2, *chn-1* and CHN-1 gf; (**d**) N2, *chn-1, hsp-43* and *hsf-1*. Data are expressed as mean (percentage of control) ± SEM

**Fig. 2:**
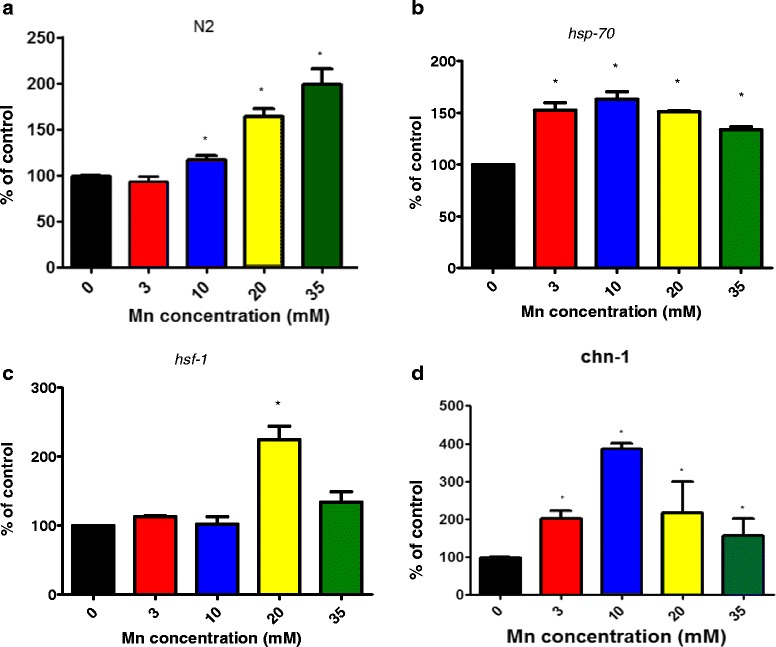
Mn-induced protein carbonylation in different *hsp* mutants. **a** N2; (**b**) *hsp-70*; (**c**) *hsf-1*; (**d**) *chn-1*. Data are expressed as mean (percentage of control) ± SEM. * indicates statistical difference from control group (*p* < 0.05)

The loss-of-function of some HSP genes led to increased sensitivity towards Mn (Table 2). While Mn-induced lethality of the transgenic strains lacking *hsp-4*, *hsp-43*, *hsf-1* and *hsp-12.6* were indistinguishable from wild type worms, *hsp-70* mutants exhibited hypersensitivity to Mn-induced lethality (LD50 = 73.08 mM) compared to N2 worms (LD50 = 46.13 mM) (Fig. 1a, *p* < 0.05). Conversely, other genetic deletions caused decreased Mn-induced lethality in comparison to wild type worms such as *hsp-3* (which has 99.1 % homology to HSPA5 from the HSP70 family, Fig. 1b) and *chn-1* (homologous to CHIP (C-terminus of Hsc70 interacting protein), Fig. 1c). In order to identify the possible underlying mechanisms, we determined the extent of Mn-induced oxidative damage through an indirect method: the measurement of protein carbonyl content. We observed that all strains showed increased carbonylation at lower Mn concentrations in comparison to N2, corroborating the findings of the survival assay (Fig. 2a, b and d). Notably, the lack of HSF-1 neither change Mn toxicity (Fig. 1d), nor increased carbonyl content (Fig. 2c).

**Table 2:** Lethal concentration 50 % for each HSP strain exposed to Mn

Strain	N2	*hsp-70*	*chn-1*	CHN-1 gf	*hsp-3*	*daf-21*	*hsp-4*	*h sp-12.6*	*hsp-43*	*h sf-1*
LD_50_ (mM)	73.08 ± 2.2	46.13 ± 2.17^a^	30.10 ± 2.74^a^	96.92 ± 2.78^a^	34.18 ± 2.83^a^	96.11 ± 3.28^a^	67.04 ± 3.027	90.40 ± 2.54^a^	72.34 ± 3.55	56.15 ± 1.68^a^

Data are expressed as mean ± SEM (*n* = 3). ^a^Indicates statistical difference from N2 worms

**Fig. 3:**
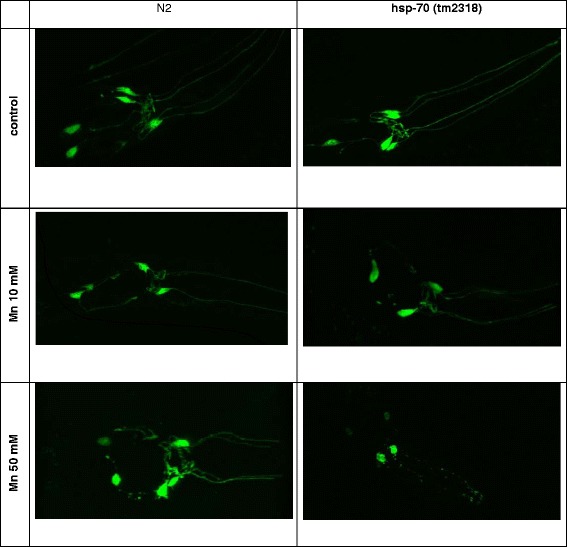
Representative images from DAergic neurons in *hsp-70 (tm2318*);p*dat-1::GFP* worms exposed to Mn at different concentrations compared to wildtype worms

**Fig. 4:**
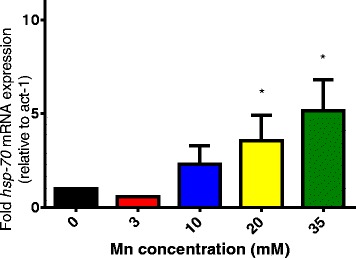
Mn exposure increases mRNA expression of *hsp-70* in *C. elegans*. Data are expressed as mean values + SEM of at least three independent experiments. * indicates statistical difference from control group (*p* < 0.05)

Considering the higher sensitivity of *hsp-70* worms following Mn exposure, we generated transgenic worms lacking *hsp-70* and expressing *pdat-1::GFP*. The expression of the green fluorescent protein (GFP) under the control of a promoter for the dopamine (DA) re-uptake transporter 1 allows the visualization of the architecture of the DAergic neurons. Following Mn exposure the *pdat-1::GFP* fluorescence and morphology of the mutants lacking *hsp-70* was compared to wild type (N2) (Fig. 3). The neurodegeneration induced by Mn in wild type worms occurred as previously reported by Benedetto et al. [27]. Remarkably, the knockout of the *hsp-70* gene caused significant degeneration in DAergic neurons following 10 mM (ballooning of the neurons soma) Mn exposure (Fig. 3). Corroborating the importance of *hsp-70* gene as a Mn-responsive gene, we further observed that the mRNA expression of this gene increases significantly with increased Mn concentrations (Fig. 4).

**Fig. 5:**
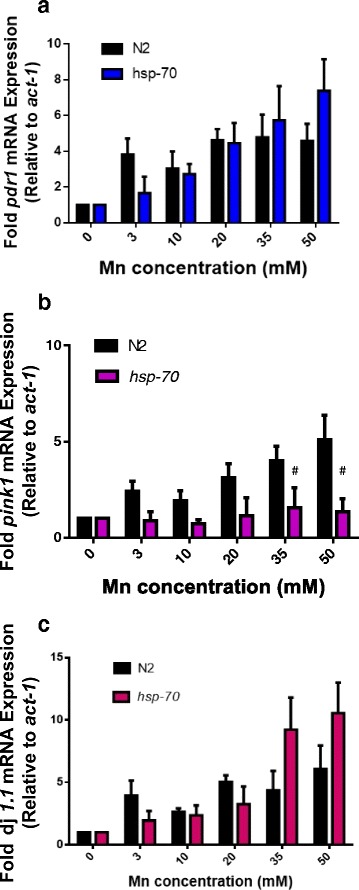
mRNA expression of PD-related genes in wild type ﻿(N2) and *hsp-70* mutants following Mn exposure. **a** *pdr-1*; (**b**) *pink-1*; (**c**) *djr-1.1*. * indicates statistical difference from control group (*p* < 0.05). # indicates statistical difference from N2 group at the same Mn concentration (*p* < 0.05)

In order to evaluate the effect of Mn exposure on the expression of PD-related genes in wild type and *hsp-70* mutants, we determined the mRNA levels of *pdr-1*, *djr-1.1* and *pink-1*. Increased Mn concentrations applied to WT worms led to a dose-dependent increase in the expression of *pdr-1, djr-1.1 and pink-1* (Fig. 5). Unexpectedly, mRNA levels of *pdr-1* and *djr-1.1* remained increased in *hsp-70* mutants following increased Mn concentrations (Fig. 5a and c). However, two-way ANOVA revealed a strong interaction between Mn concentration and genotype, supporting the notion that the dynamics of the transcriptional response to Mn exposure differs between wild type and hsp-70 worms. In particular, *hsp-70* mutation abrogates the dose-dependent increase in *pink-1* expression typically observed upon graded Mn exposure (Fig. 5b). Interestingly, this suggest that HSP-70 is specifically required for the Mn-induced increase in PINK-1 expression.

## Discussion

The cellular and molecular evolutions of most organisms rely on HSPs, which promote cell adaptation and survival under conditions of stress [2]. In the present study, we used an environmental and occupational metal toxicant and observed that the absence of *hsp-70* gene leads to increased sensitivity to Mn-induced neurotoxicity, which was associated with increased oxidative stress and specific alterations in mRNA levels of *pink-1* in *C. elegans*. Other *hsp* genes may also be related to Mn-induced neurotoxicity. However, worms lacking *hsp-4*, *hsp-43*, *hsf-1* and *hsp-12.6* were less sensitive towards Mn-induced toxicity as compared to nematodes lacking *hsp-70*.

*hsp-70* encodes a member of the HSP70 family genes in *C. elegans* with 13-members identified in the organism so far [29]. It comprises approximately 100 % homology to the human HSPA8, an isoform of heat shock cognate of 71 kDa (hsc70) [24]. The *hsp-70* gene is under normal conditions expressed constitutively throughout nematodes development. With increasing temperatures *hsp-70* mRNA synthesis is enhanced 2–6-fold [24, 30]. In mammals a large variety of cellular functions have been attributed to HSPA8 most of them through its cooperation with co-chaperones. Thereby HSPA8 participates in the chaperone-mediated autophagy, an important process that recognizes malformed proteins in order to degrade them through the ubiquitin/proteassome system [31].

Mn exposure modulates the expression of HSPs in different species [7–9, 32–35]. Most of the evidence points out to an increase of HSP-70 levels following acute Mn exposure, thus reflecting an attempt to protect from the toxic and pro-oxidative effects triggered by Mn [7–9, 32]. This is corroborated herein, as the absence of *hsp-70* led to higher vulnerability to Mn exposure, which was associated with oxidative stress, depicted by increased protein carbonylation. Observing the fluorescently labeled DAergic neurons in the outcrossed *hsp-70 (tm2318)*;*pdat-1::GFP* worms, we verified the importance of HSP-70 in protecting DAergic neurons from Mn exposure. This is in agreement with recent studies providing evidence on the important role of HSP70 in rescuing DAergic neurons in various models of PD. For instance, HSP70 suppressed α-synuclein toxicity in a transgenic *Drosophila* model of familial PD [36]. *Dong* et al. demonstrated that a Hsp70 gene transfer into DAergic neurons protect from MPTP- induced DA loss and the associated decline in DA levels in striatal mouse neurons [37]. In addition, in vitro and in vivo studies demonstrated that Hsp70 might play a role in neuroprotection against MPTP and rotenone (two models of PD) by inhibiting pro-apoptotic factors as well as by activating survival pathways [38–40]. PD-related genes DJ1, parkin and Pink1 are participating in the oxidative stress response and protect the cell against mitochondrial oxidative stressors such as Mn. Regarding the gene DJ1, the nematode expresses two orthologues named *djr-1.1* and *djr-1.2*. Recently, our group demonstrated that *pdr-1* and *djr-1.1* loss in *C. elegans* increased their susceptibility to Mn in comparison to wild type worms and that the observed enhanced oxidative stress is related to increased Mn accumulation [19]. In addition, the higher Mn accumulation caused by loss of *pdr-1/parkin* gene was due to reduction of ferroportin (a Mn cell exporter) expression in worms [20]. Furthermore, *Chen* et al. demonstrated that worms overexpressing DJR-1.2 are not subject to lifespan reduction caused by Mn exposure, in contrary to *djr-1.2* mutants [41]. Interestingly, under stress conditions, DJ-1 is translocated to mitochondria by HSP-70 [42]. It was further shown that a bcl-2 associated Athanogene 5 (BAG5) can enhance DAergic neuronal death by inhibiting both Parkin and the chaperone activity of Hsp70 [43]. Hence, we hypothesized that the absence of *hsp-70* would alter the expression of these genes. First, we verified for the first time that Mn increases mRNA levels of all these genes in wild type worms, which is in agreement with the hypothesis that these proteins are required to protect cells against Mn-induced toxicity [19, 20, 41]. While in the *hsp-70* mutants mRNA levels of *pdr-1* and *djr-1.1* increased dose-dependently in a manner indistinguishable from N2 worms, the *pink1* expression failed to increase and was not significantly different compared to non-treated mutants. *Pink1* (PTEN-induced kinase 1) is a mitochondrial kinase consisting of 581 aminoacids that encode a mitochondrial targeting sequence, a transmembrane domain and a Ser/Thr kinase domain. PINK1 is believed to confer neuroprotection by policing mitochondrial integrity [44] and a growing amount of data links dysfunction of mitochondrial dynamics with PD [45, 46]. Hence, loss of *pink-1* is associated with mitochondrial impairments, oxidative stress, and DAergic neuronal loss, as DA neurons may be particularly vulnerable to mitochondrial dysfunction [47, 48]. A proteomic study of *Triplett* et al. with PINK1 knockout mice showed that these animals have reduced HSP-70 levels in their brain [49]. Herein, we observed that *hsp-70* mutants blocked *pink-1* - mRNA expression following Mn exposure compared to the respective dose-dependent increase observed in WT worms, which reinforces the relationship between these two genes. In accordance, as we observed a significant impairment of DAergic neurons in the worms lacking *hsp-70*, we can infer that the mitochondrial dysfunction provided by Mn, plus absence of an important chaperone and the failure of the cells to increase PINK1 expression would culminate with the higher damage to these neurons. Constructing a worm that overexpress PINK1 in a *hsp-70* KO background would give us a more reliable view on the role of *pink-1* and *hsp-70* in Mn-induced DAergic degeneration.

Working with gene profiling in *C. elegans*, which can be visualized in vivo using transgenic GFP-tagged strains, *Anbalagan* et al. demonstrated that Cd^2+^, Cu^2+^, Hg^2+^ and Zn^2+^ exposure induce the heat shock genes quite strongly (*hsp-16.1, hsp-16.2, hsp-6, hsp-60* were at least 2-fold increased) [50]. Notably, we observed that Mn exposure increased mRNA levels of *hsp-70* (Fig. 4) and that HSP-4::GFP and HSP-6::GFP levels were significantly increased following Mn exposure, reinforcing the fact that this metal can indeed modulate these chaperones (data not shown).

We also observed that the absence of other chaperones as *hsp-3* and *chn-1* led to increased Mn-induced toxicity including compared to wild type woms. *hsp-3*, is expressed constitutively and is non-heat inducible; its mRNA is most abundant at the L1 larval stage [24]. Since mRNA of *hsp-3* is found at maximum levels in the L1 stage [30], it might be very important to protect the larvae against toxicants. *chn-1* is the homologue of the human CHIP, which is very important for removing defective and misfolded proteins. *Springer* et al. demonstrated that CHN-1 forms a protein complex with PDR1/Parkin, in order to ubiquitylate proteins [51]. Hence, loss of *chn-1* already causes issues in worms development [52]. Consequently, we decided not to pursue further investigations into DAergic neuronal vulnerability in these two mutants. Interestingly, loss of *hsf-1* (heat shock factor) did not cause significant vulnerability to Mn exposure (Fig. 1d). This is not surprising because it has been demonstrated that HSF-1 is not the only transcription factor that activates HSPs expression. In fact, it has been demonstrated that DAF-16 and SKN-1 can also modulate the transcription of some HSPs [53, 54].

## Conclusions

Taken together, our data suggest that Mn exposure modulates HSP expression, particularly HSP-70, in *C. elegans*. Furthermore, loss of *hsp-70* prones worms to increased protein oxidation and increased DAergic neurodegeneration following Mn exposure. This might be associated with a blockage of the pink1 expression, which can hypothetically exacerbate mitochondrial dysfunction caused by Mn exposure since *pink-1* expression is normally increased in wild type worms following Mn exposure. Consequently the current study provides evidence for the neuroprotective role of *hsp-70* in Mn-induced neurotoxicity and a possible protective role of overexpressing *hsp-70* needs to be clarified in future studies.

## Abbreviations

CHIP: C-terminus of Hsc70 interacting protein
DA: dopamine
DAT: dopamine transporter
DNPH: dinitrophenylhydrazine
DTT: dithiothreitol
GFP: green fluorescent protein
HSF: heat shock factor
HSP: heat shock protein
IgG: Immunoglobulin G
Mn: manganese
MPTP: 1-methyl-4-phenyl-1,2,3,6-tetrahydropyridine
PD: Parkinson’s Disease
Pink1: PTEN-induced kinase 1
WT: wildtype
